# Retrospective analysis of antimicrobial resistance associated with bovine respiratory disease

**DOI:** 10.1128/aem.01909-24

**Published:** 2025-02-07

**Authors:** Daniel Kos, Murray Jelinski, Antonio Ruzzini

**Affiliations:** 1 Department of Large Animal Clinical Sciences, Western College of Veterinary Medicine, University of Saskatchewan 70399 , Saskatoon, Saskatchewan, Canada; 2 Department of Veterinary Microbiology, Western College of Veterinary Medicine, University of Saskatchewan 548643 , Saskatoon, Saskatchewan, Canada; 3 Department of Biochemistry, Microbiology and Immunology, College of Medicine, University of Saskatchewan 12371 , Saskatoon, Saskatchewan, Canada; Centers for Disease Control and Prevention, Atlanta, Georgia, USA

**Keywords:** antimicrobial resistance (AMR), bovine respiratory disease (BRD), surveillance studies, genomics, metagenomics, *Mannheimia haemolytica*, *Pasteurella multocida*, *Histophilus somni*

## Abstract

The administration and utility of antibiotics to control and treat bovine respiratory disease (BRD) in beef cattle feedlots is a growing concern. Antimicrobial resistance (AMR) among BRD-associated bacterial pathogens has been the subject of cultivation-dependent and cultivation-independent surveillance. Bacterial genome sequencing and metagenomic approaches facilitate the characterization of AMR in the beef industry; however, the current collection of cattle-associated AMR research programs lack connections to each other. A more integrated view of how antimicrobial use (AMU) is related to resistance at a gene level is needed. We sought to establish a catalog of commonly observed AMR genes (ARGs) in opportunistic bacterial pathogens that contribute to BRD using publicly available data sets that were generated by the scientific community with and without AMU in mind. The presence of these clinically relevant ARGs appeared to differ by geography. Greater sampling in North America facilitated the generation of a list of ARGs often encoded by *Mannheimia haemolytica* and *Pasteurella multocida*. Detection of clinically relevant ARGs in shotgun metagenomes of cattle-associated and accessible feedlot samples such as water, soil, and feces was possible but limited by relative sequence read abundance. An exception was the tylosin esterase-encoding gene *estT*, which is among the most frequently observed ARGs in *M. haemolytica* and feedlot-related metagenomic data sets. Finally, by re-evaluating studies on the impact of AMU on AMR in beef production systems, we show that conventional practices, including in-feed antibiotic use, increase the relative abundance of ARGs in animal-derived samples.

**IMPORTANCE:**

This retrospective analysis delivers a list of ARGs found in opportunistic pathogens that contribute to BRD. The high incidence of BRD in North America is linked to the origin and implementation of metaphylaxis to mitigate detrimental animal losses at feedlots. Notably, ARGs commonly observed in these pathogens isolated in North America were not conserved across the globe, underscoring the relationship between regional AMU and AMR. A positive relationship was also observed between the relative abundance of ARGs in cattle-associated metagenomes with greater exposure to antibiotics. Overall, this analysis should help to guide future surveillance efforts and experimental designs to more directly evaluate the impacts of feedlot practices on AMR.

## INTRODUCTION

The beef industry in North America is an integrated system comprised primarily of cow-calf producers, backgrounding operations, and feedlots. Generally, calves are born in the spring and weaned in the fall. Following weaning, they are sold into feedlots where they are placed on high-energy diets and fed to slaughter. Alternatively, they may be retained by the cow-calf producers and sold the following spring as yearling cattle. These cattle are pastured on grass prior to their sale into feedlots. Calves are typically either marketed directly to feedlots or purchased from centralized auction markets. The latter is still very prevalent in western Canada, for example, and results in calves from many different sources (cow-calf producers) congregating and then being mixed, sorted, and sold as cohorts (lots) of similar bodyweight. This results in the transmission of bacteria and viruses, particularly those associated with bovine respiratory disease (BRD), which has long been described as the leading cause of morbidity and mortality in North American feedlot cattle (1). BRD is also colloquially known as “shipping fever” because the incidence of the disease markedly increases within days to weeks after animals arrive at feedlots. The onset of clinical symptoms is related to the multi-factorial nature of the disease. Specifically stress related to transport, particularly during inclement weather, combined with mixing and sorting which disrupts the calves’ social hierarchies, leads to immunosuppression and a concomitant spike in BRD cases (2).

Bacteria most commonly associated with BRD include the Mollicute *Mycoplasmopsis bovis* (formerly *Mycoplasma bovis*), and members of the *Pasteurellaceae* family, *Histophilus somni, Mannheimia haemolytica, Pasteurella multocida,* and, to a lesser extent, *Bibersteinia trehalosi* (3, 4). *Trueperella pyogenes*, a member of the family *Actinomycetaceae*, is also frequently isolated from cattle nasal swabs, lung tissue, and joint fluid at a higher frequency than other BRD pathogens, suggesting a role in pathogenesis (5). Co-occurrence of these bacteria with viral contributors to BRD (e.g., BVDV: bovine viral diarrhea virus and BRSV: bovine respiratory syncytial virus) is common (6). The bacteria are considered to be opportunistic pathogens: they typically reside in the animal’s upper respiratory tract but can spread between naïve and immunocompromised animals, resulting in clinical disease when inhaled into the lungs (7). Common BRD sequelae, which are caused by the same bacteria, include chronic pneumonia, polyarthritis, and myocarditis (8, 9).

Feedlot animals are intensively managed to help mitigate the impact of disease with metaphylactic antimicrobial use (AMU) being a common and effective strategy for controlling BRD (10). It is particularly effective for high-risk calves, where risk is assessed and defined based on the combination of factors such as age, weight, commingling, procurement method, previous vaccination, and management histories. Transportation over relatively long distances in poor weather (cold and precipitation) is an additional variable that is considered by veterinarians in developing programs to mitigate risk. Common metaphylactic agents include florfenicol, 15-membered macrolides (e.g., tulathromycin), and oxytetracycline with subsequent treatment of clinical disease by these same antibiotics as well as 16-membered macrolides (e.g., tildipirosin), fluoroquinolones, and cephalosporins (10). Accordingly, antimicrobial resistance (AMR) is a consideration when choosing antimicrobials to manage BRD. AMR can arise from intrinsic and extrinsic determinants. In the context of BRD, *M. bovis* lacks a cell wall and hence is intrinsically resistant to some drug classes (e.g., β-lactams) and has evolved resistance to other classes (e.g., macrolides and tetracyclines) via genetic mutations to their targets (11, 12). The *Pasteurellaceae* implicated in BRD are known to encode for specific AMR genes (ARGs) that have evolved roles in resistance. These ARGs belong to a diverse collection of genes that span pathogenic and non-pathogenic organisms, which collectively form the resistome (13). Due to the impact of BRD on animal health and food production, the AMR phenotypes of clinical BRD isolates is a subject of intensive research. A western Canadian feedlot study reported AMR in >95% of lung isolates with macrolide and tetracycline resistance being common (5). Contemporary BRD pathogen AMR surveillance programs have been used to integrate culture-dependent (antimicrobial susceptibility testing; AST) and culture-independent (DNA-based) methods to investigate and catalog ARGs and their mechanisms of resistance (4, 14, 15).

Whole-genome sequencing (WGS) has been used to investigate BRD-associated bacteria derived from clinically sick and healthy animals. The number of available genomes of each organism corresponds to both their clinical importance and ease of bacterial cultivation. There are >2,000 publicly available *M. haemolytica* genomes, a BRD pathogen that is frequently isolated from lung tissue and nasal swabs (5, 15). *H. somni* is associated with fatal myocarditis; however, the relatively fastidious nature of the organism has, in part, resulted in just ~70 genomes (16, 17). There are ~300 *P*. *multocida* genomes isolated from cattle, representing ~15% of the genomes for this bacterial species commonly recovered from a range of hosts (18). Surveillance of human enteropathogens associated with bovids, such as *Escherichia*, *Salmonella*, *Campylobacter,* and *Enterococcus,* has also contributed to our knowledge of food-related AMR (19–21). Finally, untargeted approaches using metagenomic sequencing of complex samples obtained from cattle and feedlots are increasingly used to investigate AMR. Examples include metagenomic sequencing of DNA isolated from feedlot soil and wastewater samples (22, 23). Metagenomic sequencing of fecal samples has also been used to characterize how AMU modifies the resistomes of cattle (24–28). The accessibility of next-generation sequencing (NGS) platforms has rapidly and dramatically shifted how AMR surveillance is conducted.

The socioeconomic impacts of AMR coupled with the emergence of cost-effective NGS has led to an increase in AMR surveillance in the North American beef cattle production. Nevertheless, pheno- and genotypic methodologies have resulted in the generation of data sets that are compartmentalized. Accordingly, we sought to provide an integrated overview of the status of AMR in BRD pathogens by reanalyzing a comprehensive suite of WGS and metagenomic sequence data. Our goal was to identify the clinically relevant ARGs associated with BRD pathogens in North America and to compare them to ARGs readily detected by metagenomic surveys. More explicitly, we aimed to categorize ARGs as clinically relevant based on their roles in protecting bacteria against approved treatments, their prevalence in BRD pathogens, as well as relative abundances in the feedlot environment. This harmonized strategy revealed a set of ARGs enriched in North American BRD pathogens. Our findings are discussed in the context of AMU, providing insight for future surveillance and molecular epidemiological studies. In particular, we highlight the prominence of *estT*, a gene that our group recently annotated as an important macrolide resistance determinant but that was overlooked for decades (29).

## RESULTS AND DISCUSSION

### Identification of clinically relevant ARGs enriched in BRD pathogens

To define the scope of AMR in BRD pathogens, 3,995 genomes were queried for the presence of well-established ARGs using the Comprehensive Antibiotic Resistance Database (CARD) Resistance Gene Identifier (RGI) tool (30). This well-curated database includes entries that are largely defined as extrinsic resistance factors that are found across disparate phyla; the analysis does not consider intrinsic resistance factors (e.g., membrane permeability and target site polymorphisms) of individual organisms from which the genomes originated. From this collection of genomes, which was largely derived from isolates from the United States (Table S1), we identified ARGs in five of the six main BRD pathogens: *B. trehalosi, H. somni, M. haemolytica, P. multocida,* and *T. pyogenes* (Table 1; Table S2). *M. bovis* was excluded because AMR is not mediated via stand-alone ARGs but rather through nucleotide polymorphisms that modify antimicrobial binding sites (31, 32). In addition, we included 81 *Fusobacterium necrophorum* and 1,028 *E. coli* O157:H7 genomes as non-BRD representatives. *E. coli* O157:H7 is a well-studied cause of foodborne illness, causing enterohemorrhagic disease in people, and has often been used as a sentinel for AMR at feedlots (33–35), whereas *F. necrophorum* causes foot rot and liver abscesses in feedlot cattle (36). Finally, 24,532 genomes from the genus *Acinetobacter*, which includes a group of environmental organisms found in the feedlot environment (37–39), were also compared to BRD pathogens. Over 99% of the *E. coli* O157:H7 and *Acinetobacter* spp. genomes contained at least one ARG compared to only 7% of *F. necrophorum* genomes. The latter feedlot pathogen also had a lower incidence of ARGs than the BRD pathogens: 75% of *H. somni*, 37% of *M. haemolytica,* and 43% of *P. multocida* encoded for at least one ARG, respectively. Despite the availability of a larger number of sequenced genomes, fewer unique ARGs were identified within *M. haemolytica* (27 unique ARGs in ~2,300 genomes) than *P. multocida* (47 unique ARGs in ~1,500 genomes). In just 66 *H*. *somni* isolates, 15 unique ARGs were identified.

**TABLE 1:** Detection of ARGs in common BRD pathogens and other sentinel bacterial species associated with cattle^*a*^

BRD pathogen	Total genomes [ARG-containing]	Cattle or feedlot-associated genomes		Unique ARG count/taxon
		North America	Non-North America	
*Histophilus somni*	66 [49]	63 [48]	1 [0]	15
*Pasteurella multocida*	1,549 [668]	73 [55]	238 [100]	47
*Mannheimia haemolytica*	2,327 [872]	2,168 [797]	131 [71]	27
*Bibersteinia trehalosi*	8 [5]	5 [4]	0	12
*Trueperella pyogenes*	35 [27]	1 [1]	11 [9]	15
Non-BRD Feedlot pathogen				
*Fusobacterium necrophorum*	81 [6]	11[3]	1[0]	8
Feedlot AMR sentinel				
*Escherichia coli* O157:H7	1,028 [1,027]	202 [202]	7 [7]	89
Environmental organisms and human pathogens				
*Acinetobacter* spp.	25,380 [25,361]	9 [9]	78 [78]	883

^*a*^ ARGs were defined by the CARD v3.3.0; CRP, a global regulator, was omitted.

The use of genomic data to define the prevalence of AMR should be considered in the context of traditional standardized phenotypic BRD surveillance. Antimicrobial susceptibility tests (ASTs) have been performed on large collections of BRD pathogens in the United States, Canada, and Germany, for example, and they have demonstrated that phenotypic resistance to antibiotics is increasing (40–42). The susceptibility of North American *M. haemolytica* isolates to tilmicosin, a common BRD treatment, for example, dropped from 90% to 60% between 2000 and 2009 (40). Another broad study from western Canada reported high recoveries of multidrug-resistant (MDR) BRD organisms, defined as those resistant to ≥4 antimicrobial classes (5). Specifically, 56% of *M. haemolytica*, 80% of *P. multocida,* and 33% of *H. somni* were deemed to be MDR based on AST data. For comparison, our CARD-based search revealed that 80% of *M. haemolytica*, 30% of *P. multocida,* and 54% of *H. somni* isolated from cattle in North America (mostly from the United States; ~94%) carried ≥4 ARGs for distinct antimicrobial classes. A more recent study reported that 86% of cattle shed MDR *M. haemolytica* after 14 days of conventional management in the United States (43). It should be noted that disparities between the results of primarily phenotypic studies and our effort to consolidate and report genotypic resistance, especially when considering multidrug resistance, may arise from additional intrinsic resistance factors that are not identified by an ARG database search.

The amount or completeness of metadata associated with the genomes limited our analysis. The origins of the isolates, be it from sick, healthy, or dead animals, were infrequently reported, and information related to treatment histories of the hosts was also absent. For example, at least 40% of *H. somni* were derived from sick or dead cattle, whereas the health status or source material for 57% of *M. haemolytica*, which has been the subject of extensive surveillance (44, 45), was missing. This missing metadata and the relatively small number of genomes limited our ability to compare bacteria based on host health status and or antimicrobial exposure. Nevertheless, we identified AMR trends that emerged at both the gene and species-levels.

**Fig 1:**
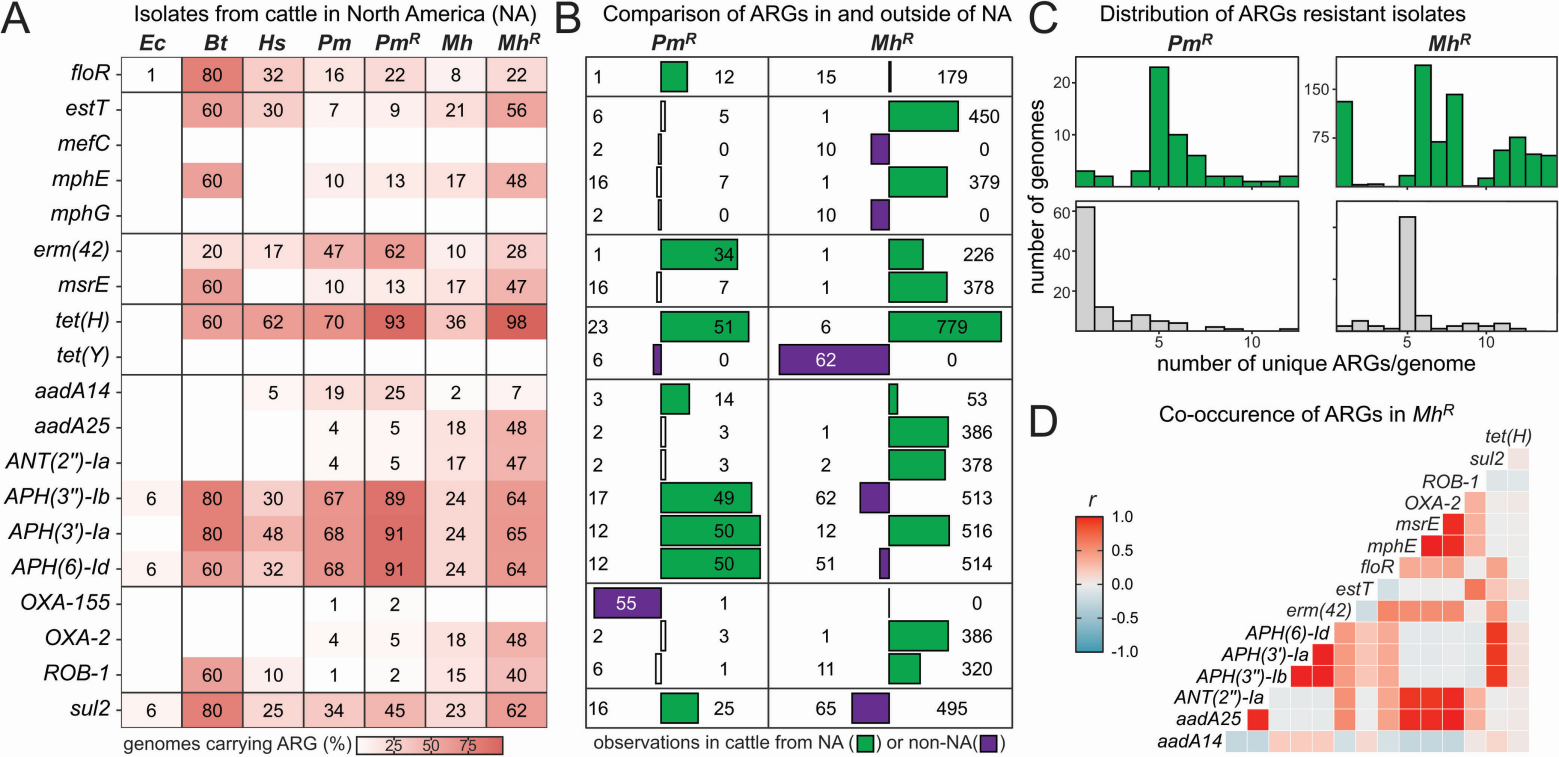
Summary of ARGs commonly observed in BRD pathogens isolated in North America. (**A**) Heatmap showing the most frequently observed antimicrobial resistance genes (ARGs) in bovine respiratory disease (BRD) pathogen genomes of North American (NA) origin. The fraction of each taxonomic group carrying the listed ARGs is shown as a percentage. Groups include *Escherichia coli* (*Ec*)*, Bibersteinia trehalosi* (*Bt*), *Histophilus somni* (*Hs*), *Pasteurella multocida* (*Pm*), and *Mannheimia haemolytica* (*Mh*). The *Pm^R^* and *Mh^R^* groups are a subset of the genomes from these taxa that encode for ≥1 ARG identified by CARD, excluding *CRP*, which was observed in all genomes. (**B**) Bar plot showing a comparison of normalized ARG frequencies in *Pm* and *Mh* from NA and non-NA origin. The number of genomes encoding specific ARGs is listed. Only ARGs in which there was >5% difference between NA and non-NA origin are shown. (**C**) Histogram showing the distribution of unique ARGs in *Pm^R^* and *Mh^R^*. Green and gray-colored histograms show NA or non-NA, respectively. (**D**) Heatmap showing the Pearson correlation coefficient (*R*) that describes the co-occurrence of pairs of commonly observed ARGs in *Mh^R^* having a prevalence of >5%.

Gene-level analysis revealed a series of frequently observed ARGs within the genomes of BRD pathogens isolated in North America (Fig. 1A). Notably, the ARGs commonly found in BRD pathogens varied greatly from *E. coli* associated with foodborne illness. Overall, our composite analysis also closely matched a recent dairy-focused report that studied BRD isolates from weaned dairy heifers (46). Many of the ARGs that were identified were reflective of industry-specific AMU, including florfenicol (*floR*), macrolides *(estT*, *mphE*, *erm(42)* , *msrE*], tetracyclines (*tet(H*)), and β-lactams (*ROB-1* and *OXA-2*). Furthermore, there were geographic differences in the enrichment of specific ARGs in AMR groups of *P. multocida* (*Pm^R^*) and *M. haemolytica* (*Mh^R^*) originating from within and outside of North America (Table 1; Fig. 1B). North American *Pm^R^* and *Mh^R^* isolates also encoded for more ARGs/genomes than their global counterparts (Fig. 1C). Resistance determinants for antimicrobials that were not industry-specific were also commonly observed. Aminoglycosides and sulfonamides are infrequently used in the cattle industry; however, aminoglycoside modifying enzymes (*aadA*, *ANT(2’’)-Ia*, *APH(3’’)-Ib*, *APH(3’)-Ia,* and *APH(6)-Id*) and a sulfonamide resistant dihydropteroate synthase (*sul2*) were frequently observed in *Pm^R^* and *Mh^R^* genomes. The observation of ARGs directly related and unrelated to AMU can be explained by the typical co-occurrent patterns of these genes in clusters. Since ARGs for different antimicrobial classes can be physically clustered together, the use of even a single antimicrobial class can provide selection for multiple ARGs appearing in multigene units that we refer to as ARG clusters.

We observed patterns of ARG co-occurrence in *M. haemolytica*. A correlation analysis using *Mh^R^,* the largest group of AMR pathogens in the collection (*n* = 872 for North America), demonstrated co-occurrence of expected and unexpected ARGs based on conventional feedlot AMU (Fig. 1D). The macrolide phosphotransferase encoded by *mphE* co-occurred with *msrE* (Pearson correlation coefficient*, r* = 1.0), which confers ribosomal protection against the same class of antibiotic. These genes also co-occurred with two aminoglycoside nucleotidyltransferases (*aadA25* and *ANT(2’’)-Ia, r* = 1.0 and 1.0). These correlations are consistent with ARG clusters embedded in integrative and conjugative elements (ICEs) and plasmids (6, 47). Indeed, ARG clusters in members of both the *Mh^R^* and *Pm^R^* groups have been reported in studies of ICEs (48, 49) and epidemiological work has suggested that feedlots, countries, and veterinary prescribing practices are correlated to AMR profiles of *Pasteurellaceae* (50).

### Identification of mutations conferring resistance to fluoroquinolones

Target-site mutations often mediate resistance to macrolides (51), tetracyclines (52), and fluoroquinolones (53). Genes that have evolved to confer fluoroquinolone resistance (e.g., *qnr* genes) were not identified in BRD pathogens; however, enrofloxacin is used to treat BRD and resistance has been reported. Thus, we supplemented our CARD-driven approach with a search for nucleotide polymorphisms in *M. haemolytica* isolated from cattle (*n* = 2,299) since the phenomenon is well-studied in these organisms. Missense mutations to type II and type IV DNA topoisomerases *gyrA* and *parC* are known to participate in resistance to fluoroquinolones in these bacteria (43, 54–57). More specifically, phenotypic resistance is associated with three co-occurrent missense mutations at GyrA Ser83, GyrA Asp87, and ParC Glu97 (Table 2; Table S3). The three co-occurrent mutations were observed in 395 *M*. *haemolytica* genomes (17%), suggesting phenotypic resistance at a level similar those inferred for other relevant antibiotics based on CARD gene finding (Fig. 1A). Additionally, all 395 mutants also encoded for at least one canonical ARG identified using the CARD. In other words, nearly half (46%) of the *Mh*^R^ group carry resistance mutations for the fluoroquinolones. While co-occurrence of all three mutations has been linked to the development of phenotypic resistance in *M. haemolytica*, the proportions of mutations were not equivalent across all three sites in the published genomes. Additional sites have been implicated in fluoroquinolone resistance in other organisms (53), including *P. multocida* (58); however, these mutations require additional molecular microbiological studies and matched geno- and phenotyping in BRD-associated bacteria to understand their role in AMR.

**TABLE 2:** Detection of mutations known to confer fluoroquinolone resistance in cattle-associated *Mannheimia haemolytica* (*n* = 2,299)

Gene	Amino acid substitution (codon change)	No. of genomes (proportion; %)
*gyrA*	S83F (TCT > TTT)	545 (23.7%)
	S83Y (TCT > TAT)	18 (0.8%)
*gyrA*	D87N (GAC > AAC)	398 (17.3%)
	D87H (GAC > CAC)	55 (2.4%)
	D87G (GAC > GGC)	16 (0.7%)
	D87Y (GAC > TAC)	7 (0.3%)
*parC*	E89K (GAA > AAA)	400 (17.4%)

### Detection of inactivated ARGs in BRD pathogens

ARG detection is not equivalent to AMR protein detection. In fact, bioinformatically pairing ARG detection at the DNA and protein levels revealed a cryptic population of resistance determinants: genes that are silenced through phase variation and other minor mutations. A total of 10 inactivation events in the *Mh^R^* and *Pm^R^* genomes were detected through manual inspection of DNA and protein-based searches (Table 3). This included frameshift mutations to *erm(42) , tet(H), floR, sul2,* and *APH(3’’)-Ib* via homopolymer variation as well as the direct substitution of a codon for a premature stop sequence. Proportionally, intragenic inactivation was greater in *P. multocida* with ~2.4% of *tet(H*)-identified sequences being subject to inactivation. Similar observations and frequencies of intragenic ARG inactivation have previously been reported for MDR *Staphylococcus aureus*, including reversion of the so called silencing of AMR by mutation (SARM) phenomena (59). Further studies are required to validate SARM as a means of ARG expression regulation in *M. haemolytica* and *P. multocida* though variations in homopolymer tracts have been reported to regulate iron-related proteins in the former (60) and a galactosyltransferase encoded by the latter (61). In general, these genetic means of ARG inactivation may explain incongruencies between PCR-based surveillance, AST profiles, and treatment outcomes (59, 62, 63).

**TABLE 3:** Informatic observations of intragenic inactivation of ARGs

ARG	No. of inactivated genes (%)		Mechanism of inactivation
	*Mannheimia hemolytica*	*Pasteurella multocida*	
*erm* (*42*)	2/227 (0.9%)	1/61 (1.6%)	Frame shift via homopolymer
*floR*	0/194	1/77 (1.3%)	Codon mutation to STOP
*tet(H*)	0/788	3/124 (2.4%)	Codon mutation to STOP
*sul2*	0/562	2/211 (0.9%)	Frame shift via homopolymer
*APH(3’’)-Ib*	0/577	1/173 (0.6%)	Frame shift via homopolymer

### ARG identification in metagenomes of varied cattle-associated origins

To revisit AMR associated with cattle microbiomes and to anticipate common findings within and between metagenomic surveillance efforts, we reanalyzed 52 NCBI BioProjects that contained 879 individual sequencing read records (SRRs) of which 770 possessed reliably detectable ARGs (Tables S4 to S7). This included 12 distinct sample types of environmental and clinical origin. It is worth noting that these data sets are from standalone works (22–27, 64–76) and that the design of these individual studies differs from our attempt to repurpose DNA sequence data sets for AMR surveillance. We queried these data sets for ARGs and then assessed each as a reservoir of those identified in common participants of BRD.

**Fig 2:**
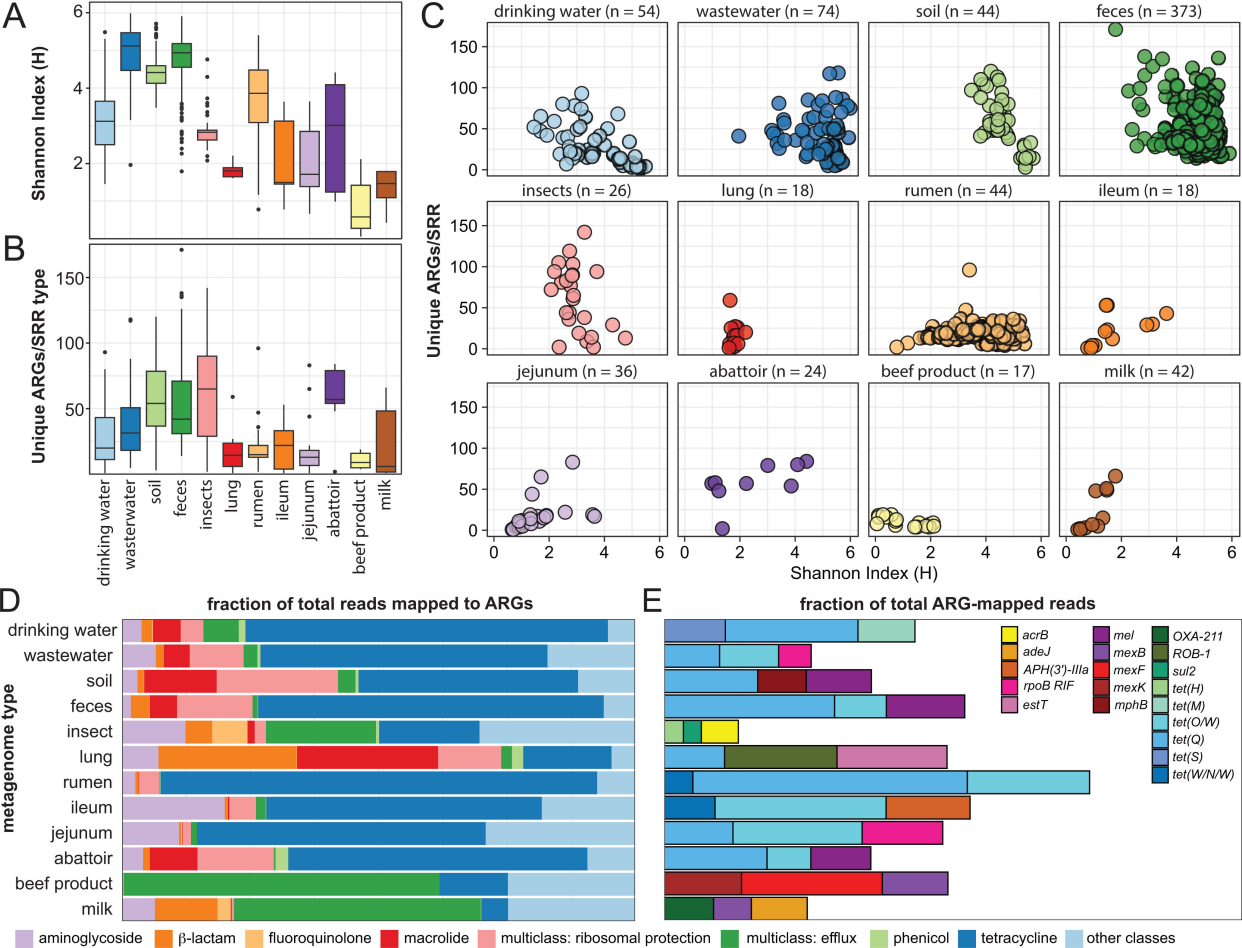
Overview of ARG detection within cattle-related metagenomes. (**A**) Shannon Index (*H*) of bacterial alpha diversity of each metagenome type. (**B**) Unique ARGs detected within sequence read record (SRR) data set types (metagenome types). (**C**) Plots comparing unique ARGs to the alpha-diversity measured for the bacterial reads within each individual SRR. (**D**) Summary of ARGs identified in metagenomes by class based on read mapping. (**E**) The top three ARGs detected in each metagenome sample type based on relative abundance of mapped reads to all detected ARGs.

The proportion and identities of bacterial reads obtained from complex biological samples varied by type. This was reflected in their Shannon indices (*H*), a measure of bacterial population alpha-diversity. Wastewater and milk metagenomes had the highest (4.9) and lowest (0.7) mean *H* indices, respectively (Fig. 2A). Fecal samples had relatively high *H* indices, whereas low diversity was observed in bronchoalveolar lavage samples of cattle that died of BRD. This observation is also corroborated by previous studies showing a decrease in bacterial diversity in cattle impacted by BRD (77, 78). The size of the resistomes varied between and within sample types (Fig. 2B). The lung (*n* = 18) and rumen (*n* = 152) contained ~13 and ~18 ARGs/sample, which were relatively small in comparison to the ARG diversity of the *Mh^R^* and *Pm^R^* data sets. ARG identification from drinking water, wastewater, feces, and insects was greater with respect to number of unique ARGs identified; however, these experiments appeared to be limited, at least in part, by sequencing depth. The number of unique ARGs for these sample types did not converge even in the largest data sets (Fig. S1). This is considered a general shortcoming of shotgun metagenomic sequencing. Thus, DNA enrichment technologies using industry-specific panels of ARGs (e.g., probe capture for genes presented in Fig. 1) should be considered in order to overcome this detection limitation for targeted studies.

The feedlot-associated resistomes reflected the major antibiotic classes used in this sector of agriculture. We identified 541 unique ARGs across the 770 SRRs investigated (Table S7). This number is comparable to the 319 resistance determinants identified in a smaller data set comprising soil, feces, water, abattoir, and vehicle samples (22). There was a notable detection of ARGs responsible for resistance to tetracyclines and macrolides, which have historical applications as feed additives, and are consistently reported by feedlot-associated AMR surveys (27, 67). Tetracycline ARGs comprise the major constituents of the known resistomes of nearly all metagenome types (Fig. 2D). Identification of ribosomal protection protein genes dominated these cattle-associated resistomes with *tet(Q*) identified among the top three ARGs in 8 of the 12 distinct sample types, and accounting for >40% of ARG reads in the rumen (Fig. 2E). Indeed, *tet(Q*) is well-known to be widely distributed in bacteria and is associated with ICEs (79). Finally, among the samples included in this retrospective analysis, the metagenomes derived from food products differed considerably from the others in their enrichment for multiclass efflux pumps. Thus, the pool of ARGs detected in food products is not tightly coupled to those detected in the feedlot environment and animal microbiome.

**Fig 3:**
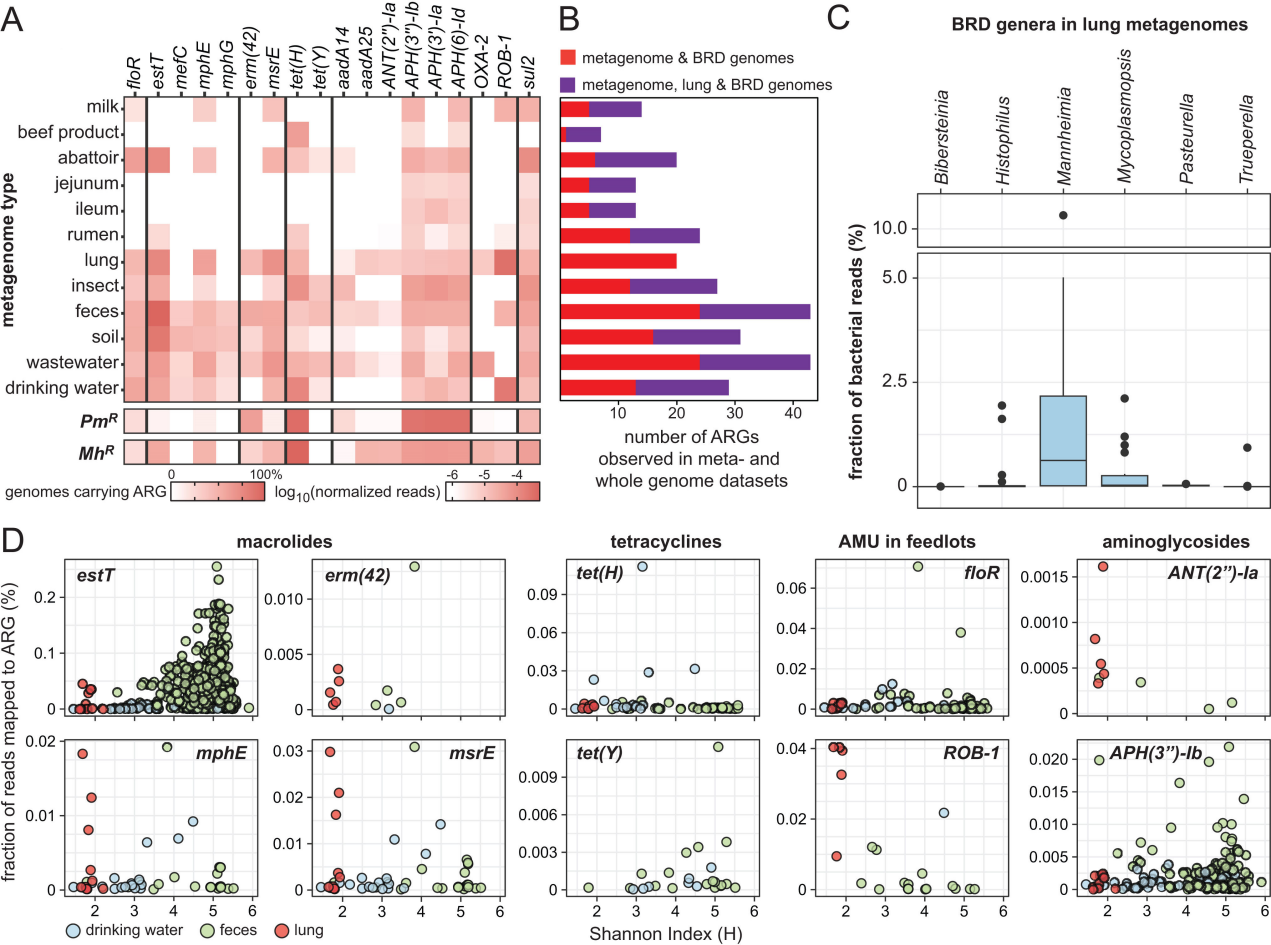
Comparison of ARGs commonly observed in BRD pathogens and metagenomic data sets. (**A**) Heatmap showing the relative abundance of BRD pathogen-associated ARGs observed in metagenomic data sets. The observed frequency in *Pm^R^* and *Mh^R^* is provided for comparison. (**B**) Stacked bar plot showing the number of ARGs found in both a given metagenome and BRD pathogen genomes (red) or within a given metagenome, lung, and BRD pathogen genomes (purple). (**C**) BRD pathogen detection in lung metagenomes. (**D**) Representative examples showing the detection of ARGs in metagenomes. Genes were selected based on their presence in BRD pathogen genomes in and outside of North America. For water, fecal, and lung metagenomes, the fraction of reads mapped to the representative ARGs are plotted against the Shannon diversity index calculated for individual data sets.

Next, we assessed how well each cattle-associated sample type might be used to predict clinical AMR. First, we analyzed how many sequences from each metagenomic sample could be mapped to frequently observed ARGs in *M. haemolytica* and *P. multocida* (Fig. 3A). Feces, soil, and wastewater showed the greatest number of detectable ARGs that overlapped with the clinically relevant lung samples and BRD genomes (Fig. 3B; Table S8). This comparison, however, is limited by the fact that *M. haemolytica* was the most abundant pathogen in the relatively few lung samples (Fig. 3C). Accordingly, the expected patterns of *M. haemolytica* ARG co-occurrences are borne out in the lung metagenomes (Fig. 3D). For example, *estT* and *ROB-1* were among the most frequently detected ARGs in the lung, which is well-aligned with their co-occurrence (*r* = 0.6) in *M. haemolytica* genomes. Likewise, *mphE*, *msrE,* and *ANT(2’’)-Ia* have similar distributions in the lung and BRD genomes (*r* = 0.9–1.0). The relative abundance of certain genes such as *estT* and *APH(3’’)-Ib* in samples such as drinking water and feces reveals the complexity in predicting clinically relevant AMR in feedlots. Here, the pre-eminent example is *estT*, which is among the most frequently detected ARGs in both pathogens and metagenomes. This widespread distribution within a range of pathogenic and environmental organisms (29) is likely to challenge the prognostic value of non-quantitative and context-independent ARG detection offered by most metagenomic surveys. Approaches that offer greater genetic context may help to resolve the taxonomic identification of clinically relevant ARGs. For example, proximity-based ligation methods have helped to resolve ARG-carrying plasmid:host pairs within wastewater communities (80). Long-read sequence platforms are also available but suffer from similar detection limitation (see below), leaving quantitative measures to established qPCR pipelines and/or DNA capture technologies.

### The impacts of AMU on the relative abundance of ARGs

The impacts AMU are multifactorial and few attempts to directly monitor them on conventional (CONV) feedlot practices have been completed. To this end, there are challenges in that CONV practices cover a variety of AMU protocols, which may include in-feed delivery and or the subcutaneous injection of metaphylactic agents and other therapeutics. The classes of antimicrobials also vary, and more than one class can be used for each of the aforementioned applications: oxytetracycline and tylosin are common feed additives, florfenicol and tulathromycin are frequently used for metaphylaxis, and tildipirosin and ceftiofur are used as first- and secondary treatments of BRD. Recently, the use of readily cultivable sentinel organisms to asses impacts of AMU on AMR (81, 82) has been replaced or complemented by culture-independent applications of NGS technologies (24–27, 67). Generally, these reports show increased relative abundances of ARGs involved in resistance to macrolides and tetracyclines (24, 26, 27). More broadly, the results have aggregated conclusions at the antibiotic class level rather than gene level.

**Fig 4:**
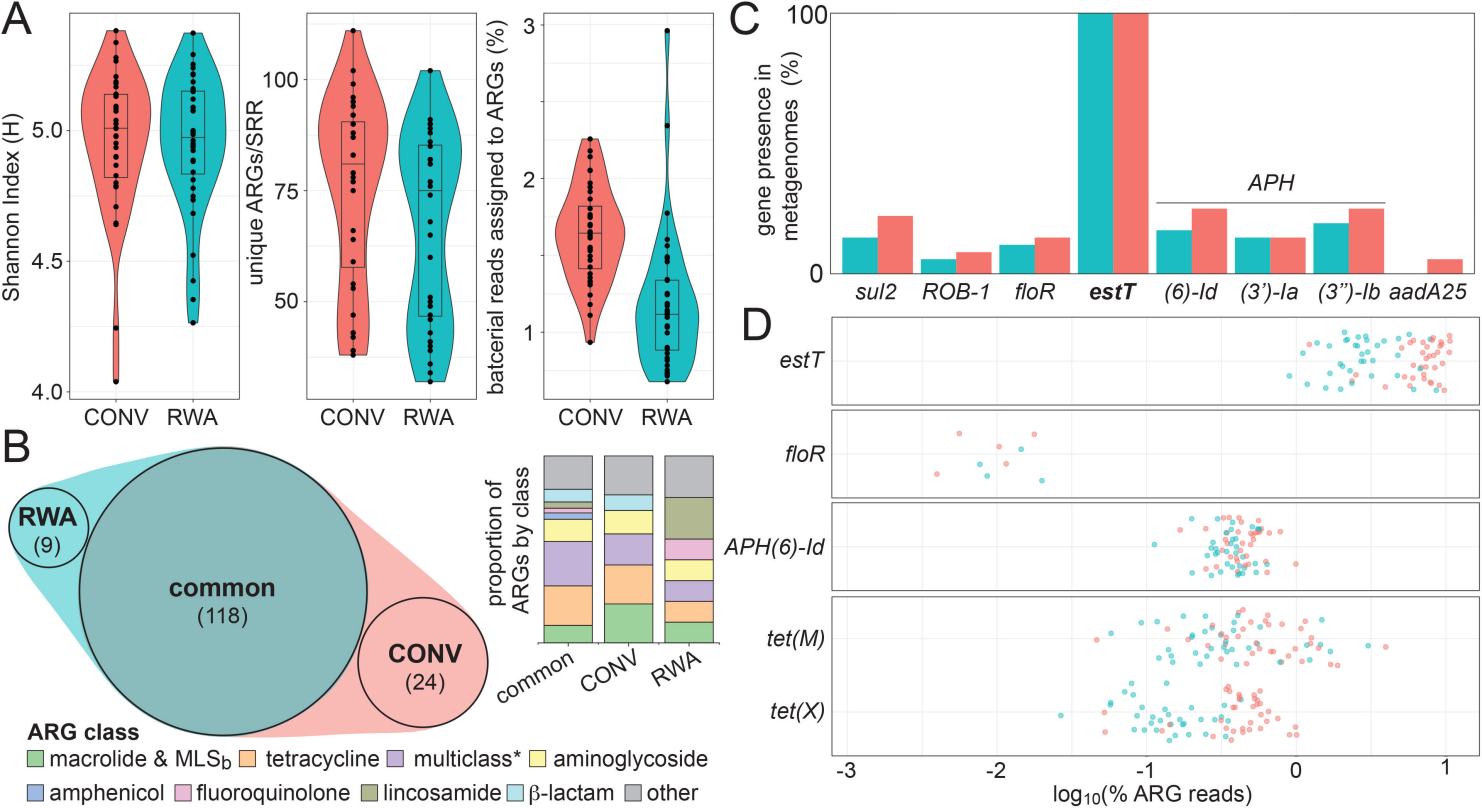
Comparison of microbiomes of animals conventionally raised or raised without antibiotics. (**A**) Overview of bacterial alpha-diversity, represented by Shannon index values (left panel), and AMR, represented by unique ARGs/sample type and the proportion of bacterial reads assigned to ARGs (middle and right panels) in CONV and RWA metagenomes. (**B**) Venn diagram showing the number of ARGs detected in CONV and RWA data sets. A pie chart shows the ARG classes observed uniquely in the CONV data sets. An asterisk denotes that multiclass resistance genes were primarily export systems. (**C**) Detection of ARGs found to be enriched in *Mh^R^* in CONV and RWA metagenomes. (**D**) Representative jitter plots showing the fraction of reads mapping to specific ARG of interests compared to the total pool of ARG reads in CONV and RWA metagenomes.

To complement our efforts to define ARGs commonly found in feedlot pathogens, we reanalyzed two studies with adequate metadata to determine the potential impacts of AMU and AMR. The first study compared fecal microbiomes from animal’s subjected to CONV feedlot practices to those raised without antibiotics (RWA) over a year period (27). While specifics related to AMU were not considered for animals in the CONV group, the RWA group was reported as being free of AMU. We began by performing high-level taxonomic analyses and matching reads to genes available in the CARD to assess differences between groups. The alpha-diversity of the communities was similar between groups. Differences between groups were found in the greater numbers of unique ARGs per Sequence Read Record (SRR; AGR/SRR) and higher relative abundances of ARGs in the CONV data sets (Fig. 4A). A total of 151 ARGs were detected across all samples, revealing a shared catalog of ARGs in CONV and RWA samples of clinical relevance: 118 were found at least once in both CONV and RWA data sets while 24 or 9 ARGs were found exclusively in the CONV or RWA groups, respectively (Fig. 4B). Closer inspection of the CONV-specific ARGs showed a slight enrichment in the proportion of macrolide resistance determinants, whereas lincosamide resistance genes were unexpectedly a defining characteristic of samples from RWA animals (Fig. 4B, inset). While lincosamides have veterinary applications (e.g., swine, cats, and honeybees), they are not labeled for use in cattle, and none were expected to be used in animals RWA. Additional studies are required, across difference geographic regions, to better assess the impacts of AMU on the resistomes of cattle.

The CONV and RWA-sourced samples differed significantly in their relative ARG abundances (Fig. 4C). This is best illustrated by *estT;* although it was found in all samples, it is present in much higher abundance in CONV feedlots (Fig. 4D). This trend was also apparent for tetracycline ARGs such as *tet(M*), a ribosomal protection protein, and *tet(X*), a flavin-dependent monooxygenase (27), which are not commonly found in organisms of clinical importance but are present in most metagenomic samples. The co-occurrence of the *estT* and *tet(X*) genes, especially on mobile genetic elements such as plasmids (83), is likely responsible for the relatively high abundance of the latter. Other clinically relevant ARGs such as *floR*, a florfenicol exporter, were sparsely detected, whereas ARGs not directly related to feedlot AMU such as the aminoglycoside phosphotransferase gene *APH6-Id* were commonly detected and were found more frequently in CONV than RWA metagenomes. The low relative abundance of *floR* may reflect the limited use of florfenicol in the study group as it contrasts with a positive association seen in a feedlot that employs the drug (39). These selected examples illustrate the impacts of feedlot practices on AMR; however, we are cautious not to overinterpret the results. Information on AMU in CONV feedlots including the numbers and identities of antimicrobial classes (e.g., florfenicol use) was and is typically not collected in feedlot-related studies. The absence of this metadata limits our interpretation beyond the most obvious observation that AMU increases the presence of ARGs in fecal microbiomes. Importantly, this interpretation differs from the initial report that aggregate abundances of ARGs did not vary across CONV and RWA data. Previously, cumulative sum scaling (84) was employed in the CONV and RWA analysis (27). Instead, we employed a straightforward, model-free, approach by normalizing ARG reads to the total number of bacterial reads within each sample. This simple approach does not make any assumptions about the status of any gene within or between any given samples, which in this case varied greatly in their bacterial contents (Fig. S2).

**Fig 5:**
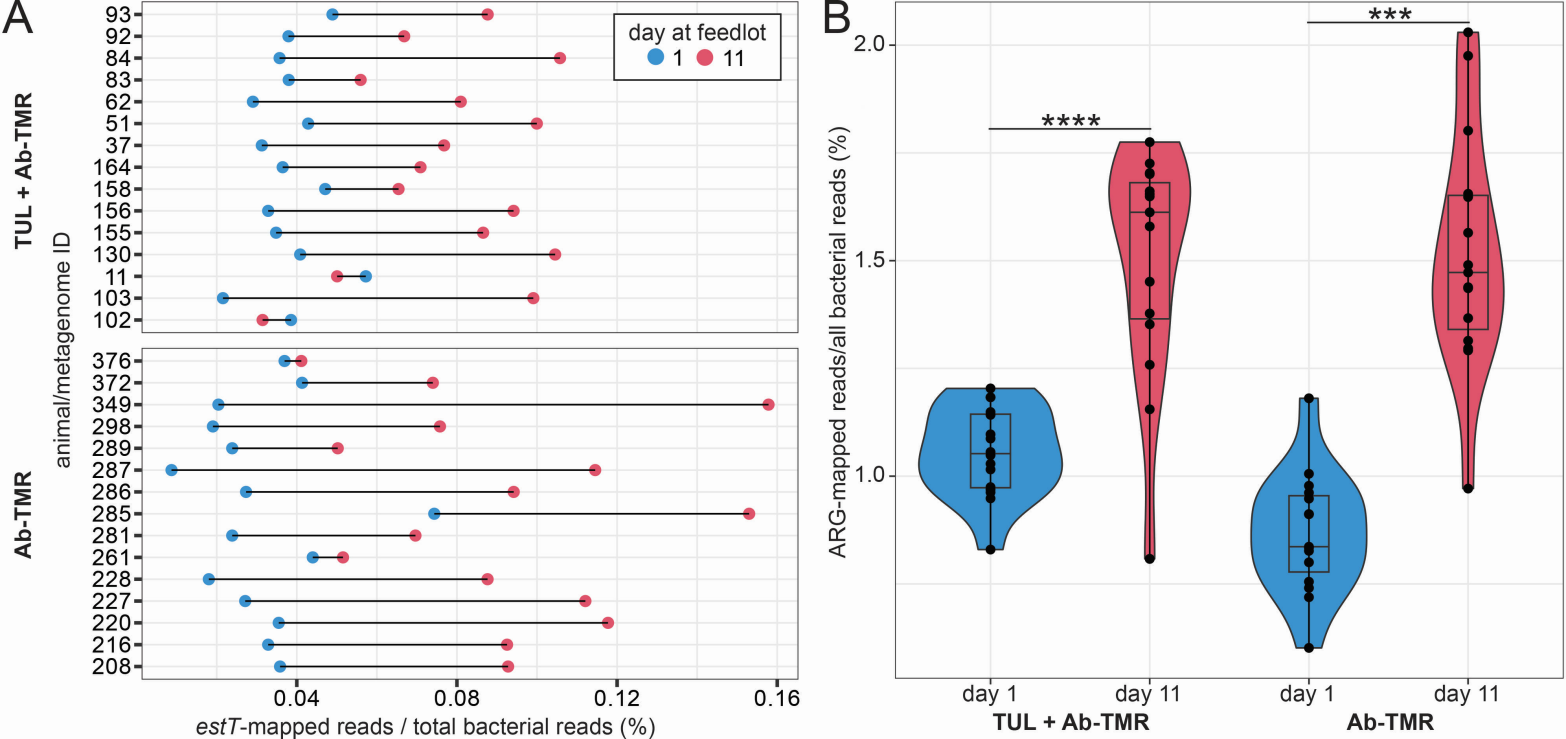
Observation of ARG-level impacts of common feedlot practices. (**A**) Changes in the relative abundance of *estT* and (**B**) total ARG reads normalized to all bacterial reads in the fecal microbiome of feedlot cattle upon and 11 days after arrival. Two experimental groups of single sourced cattle differed in that they did or did not receive metaphylaxis via subcutaneous injection of 800 mg tulathromycin on arrival. All animals were fed tylosin in the TMR.

While limited metadata was collected during the comparison of CONV and RWA practices, a recent attempt to study the impact of tulathromycin-based metaphylaxis on the ARGs present in the cattle microbiomes was conducted that included animal-level sampling and data collection (26). Interestingly, tylosin and monensin were included in the total mixed ration (TMR) provided to all animals in the study, with sampling performed both before (day 1, feedlot arrival) and after (day 11) subcutaneous administration of tulathromycin in the form of Draxxin. Thus, this study provided an opportunity to directly investigate changes to the relative abundance of *estT* in individuals that were fed tylosin as a component of an antibiotic-supplemented TMR (Ab-TMR). Importantly, the animals were single-sourced and backgrounded, limiting disease and further antibiotic intervention. Antibiotic exposures in the studied animals were, therefore, through feed or metaphylactic injection only. In fact, we considered that the inclusion of an Ab-TMR was at least equally meaningful to whether or not animals received a metaphylactic dose of tulathromycin, which the authors aimed to study. In all but 2 of 30 studied cases, the relative abundance of the *estT* gene in fecal microbiomes had increased after 10 days on a diet that included tylosin (Fig. 5A). While we expected that in-feed tylosin would select for bacteria carrying the *estT* gene, we noted that the aggregate of all detected ARGs was also more abundant at day 11 compared to day 1 (Fig. 5B). This study lacked a control group defined by an antibiotic-free TMR; however, the results – increased relative abundances of ARGs after animal arrival at feedlots – are supported by prior works that claim environmental factors, management and feeding practices impact the resistome of cattle (22, 50, 67, 85).

There have been mixed reports on the impact of in-feed antibiotics with respect to AMR in cattle. For example, in-feed chlortetracycline was reported to increase the relative abundance of tetracycline ARGs (67), whereas the effect of tylosin was reported to be statistically insignificant across geographically distinct feedlots (25). In combination, tylosin and monensin were observed to alter the cattle gastrointestinal microbiome in a manner that was consistent with antimicrobial activities (85). And, a reduction in genetic diversity associated with AMR was used to argue for AMU-based selection throughout beef production (22). In general, we note that the statistical analyses have largely been performed on collections of genes identified by various platforms characterized by variable levels of data curation. Furthermore, normalization methodology varies across studies, and these choices have strengths, weakness, and variable applicability that can affect interpretation (86). To date, the results have been conservative interpretations of the impact of AMU on AMR in feedlots. An integrated and collaborative effort is required to better evaluate the feedlot- and industry-specific consequences of AMU as well as their wider spanning impacts on public and environmental health.

### Outlook

Our retrospective analysis demonstrates that different practices can impact AMR in food production systems. An overarching limitation of this analysis was its basic reliance on publicly-available data, preventing a more complete re-examination of past experiments. Missing labels that may have allowed differentiation of data sets is a systemic issue. Nevertheless, we were able to establish sets of clinically relevant ARGs in *M. haemolytica* and *P. multocida* for North American feedlots. Proportionally, more MDR bacteria were isolated and characterized from North America than other parts of the world, which may reflect management practices and AMU. Additional data to define more fastidious and poorly studied contributors to BRD are needed to extend our knowledge and apply it to surveillance programs and the design of more rapid tests for AMR. Remarkably, we noted that *estT,* a recently discovered ARG, was among the most relevant in clinical isolates, microbiomes and industry-associated metagenomes.

**Fig 6:**
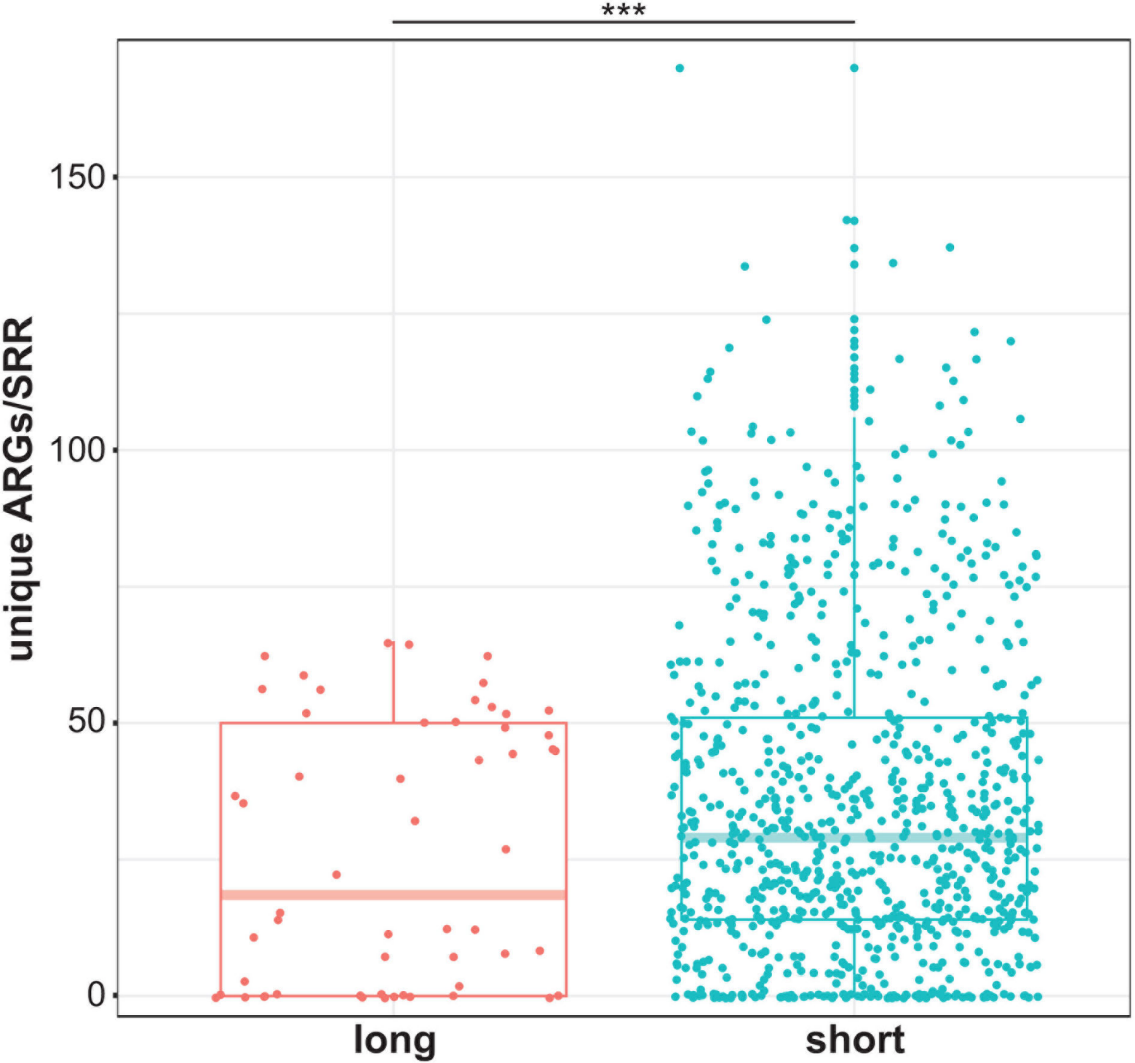
A comparison of ARG detection from long- and short-read shotgun metagenomic SRRs. A significant difference in the mean number of unique ARGs/SRR type was observed (Welch Two Sample *t*-test: *t* = −2.90, df = 66.12, *P* = 0.005).

ARG detection from complex samples is a sequencing platform-independent challenge. While we focused on short-read shotgun metagenomic data sets, long-read analyses exist (28) though they too suffer from the same lack of sensitivity. On average, short-read shotgun approaches tend to outperform long-read alternatives to feedlot AMR surveillance (Fig. 6). Thus, employing DNA hybridization-based capture probes to target ARGs should be considered an alternative to shotgun approaches. In the context of AMR, probe capture methods based on the CARD were first applied and validated using human clinical isolates and stool samples (87), and these approaches have since been extended to retail foods (88, 89). The beef cattle industry and health sector-specific designs could be adopted as platforms for future AMR surveillance and epidemiology.

## MATERIALS AND METHODS

### Detection of ARGs within BRD genomes

All genomes in NCBI GenBank belonging to *Bibersteinia trehalosi* (*n* = 8)*, Histophilus somni* (*n* = 66)*, Mannheimia haemolytica* (*n* = 2,327)*, Pasteurella multocida* (*n* = 1,549), and *Trueperella pyogenes* (*n* = 35) were retrieved (April 2024) using NCBI data sets command line tools (v15.16.1) (90). Additionally, all genomes from the genera *Acinetobacter* (*n* = 24,532) and from *Fusobacterium necrophorum* (*n* = 81) and *Escherichia coli* O157:H7 (*n* = 1,028) were also included in the analysis. The Comprehensive Antibiotic Resistance Database (CARD v3.3.0; fetched September 6, 2023) Resistance Gene Identifier (RGI, v6.0.2) was used to detect ARGs within the genomes, with nudges included (30). NCBI Entrez was used to retrieve BioSample information. Data transformation and graphing were performed in R (v4.3.1). Matches with lower than 80% identity and coverage of the gene were automatically discarded; those above 80% were inspected, showing that the vast majority met even higher matching criteria (Fig. S3). All ARGs are referenced by their “CARD Short names.”

### Manual curation and ARG counting

After the detection of unique sequences based on the CARD, we elected to collapse ARGs with >95% nucleotide sequence identity into singletons (Table S9). Sequences with >95% nucleotide identity were identified by performing an all-by-all blastn analysis of the CARD genes. For example, 13 differently numbered *FOX* genes encoding for a β-lactamase were identified but counted as a single ARG (*FOX-1*) based on the aforementioned 95% sequence identity cut-off. In these cases, we maintained the use of the CARD short name, keeping the lowest numbered homolog in a larger group sharing >95% identity. In contrast, our total counts include multicomponent systems that were counted by gene rather than as a combined resistance determinant. This is typical for database-driven AMR surveys. Thus, the genes *mexA* and *mexB* of the MexAB-OprM efflux pump are counted individually: each is a unique ARG but not uniquely a determinant of AMR.

### Detection of putative ARG inactivation events

For the *P. multocida* and *M. haemolytica* genomes in the study, a search of inactivated ARGs was performed. Mismatches between the ARGs detected by RGI, as described above, and ARGs detected through a local blastn search using the same reference database were compiled and subsequently subjected to manual inspection for mutations disrupting translation of the coding sequence was carried out and visualized using Geneious Prime 2025.03.

### Detection of nucleotide polymorphisms associated with fluoroquinolone resistance

To find nucleotide polymorphisms of interest within *M. haemolytica,* a local blastn search was performed using *gyrA* and *parC* from *M. haemolytica* (GCF_002285575.1) as the query for a database of 2,299 *Bos taurus*-associated *M. haemolytica* genomes. Seqkit (91) was used to extract the identified genes, which were aligned to the reference genome to identify polymorphism using the “Find variations and SNPs” tool within within Geneious Prime 2025.0. These results were imported into R, where it was verified that only copy per genome was extracted and aligned.

### Metagenomic sequence analyses

The NCBI BioProject list browser was used to identify and collect data metagenomic studies with available raw sequence reads. Search terms were Feedlot, Cattle, Beef, *Bos taurus*, Bovine, and Dairy. BioProjects were then manually curated and excluded if the description indicated that data were generated from other livestock or livestock facilities other than those for *Bos taurus* or if the data type was incompatible (i.e., amplified loci such as 16S rRNA sequencing or sequencing of isolates). The RGI bwt tool (30) was used with bowtie2 (92) on the metagenomes of the 52 BioProjects. Normalization of reads and taxonomic assignment was based upon read categorizing performed with Kraken2 (93) (v. 2.1.2, database constructed of Refseq bacterial reads). Completely mapped reads from RGI bwt detection were divided by the total amount of reads assigned to the bacterial clade with Kraken2 within each respective metagenome. A Venn diagram of the overlap of unique ARGs between sample types was generated using nVennR (v0.2.3) (94).

For taxonomic assignment, Kraken2 reports were processed through bracken (v. 2.7, genus level taxonomic assignment, threshold 0, 35 kmer, 100 length) (95). Shannon indices were calculated from the taxonomic assignment of reads from bracken. The total reads mapping to bacteria in each SRR was used to calculate the proportion of reads that mapped to each taxa in each SRR. Subsequently, the Shannon index was calculated with the following formula H = -∑(proportion * ln(proportion)) in R for each SRR.

To compare long- and short-read sequencing data with respect to ARG detection, the RGI BWT output of SRRs from BioProject PRJNA1096931 was compared to the cumulative outputs of the RGI bwt results from of the short-read SRRs included in this study.

### Statistical analyses

The Pearson correlation coefficient of ARGs within *M. haemolytica* genomes was performed using GGally (v2.2.1) (96). Student’s/Welch’s *t*-tests were performed paired and unpaired as appropriate considering sample origins (available in the metadata) and previous study designs.

## SUPPLEMENTAL MATERIAL

Supplemental figures aem.01909-24-s0001.pdf Figures S1 to S3.

Supplemental tables aem.01909-24-s0002.xlsx Tables S1 to S9.

ASM does not own the copyrights to Supplemental Material that may be linked to, or accessed through, an article. The authors have granted ASM a non-exclusive, world-wide license to publish the Supplemental Material files. Please contact the corresponding author directly for reuse.
