# Supplementary material for: Retrospective analysis of antimicrobial resistance associated with bovine respiratory disease
Source: Appl Environ Microbiol. 2025 Feb 7;91(3):e01909-24. doi: 10.1128/aem.01909-24 (PMC11921372; doi:10.1128/aem.01909-24)
Supplement: Supplemental figures — Figures S1 to S3. [file aem.01909-24-s0001.pdf]

Supplemental Figures

for

Retrospective analysis of antimicrobial resistance associated with bovine respiratory disease

Daniel Kos[1], Murray Jelinski[1] and Antonio Ruzzini[2,3,*]

[1]Department of Large Animal Clinical Sciences, Western College of Veterinary Medicine, University of Saskatchewan, Saskatoon, SK, S7N 5B4

[2]Department of Veterinary Microbiology, Western College of Veterinary Medicine, University of Saskatchewan, Saskatoon, SK, S7N 5B4

[3]Department of Biochemistry, Microbiology and Immunology, College of Medicine, University of Saskatchewan, Saskatoon, SK, S7N 5E5

Figure S1. Plots of unique ARGs identified compared to sequence data within each SRR S2
Figure S2. Bar plot showing the percentage of reads mapped to Bacteria by metagenome type S3
Figure S3. Histograms showing the number and distribution of ARGs identified in this study S4

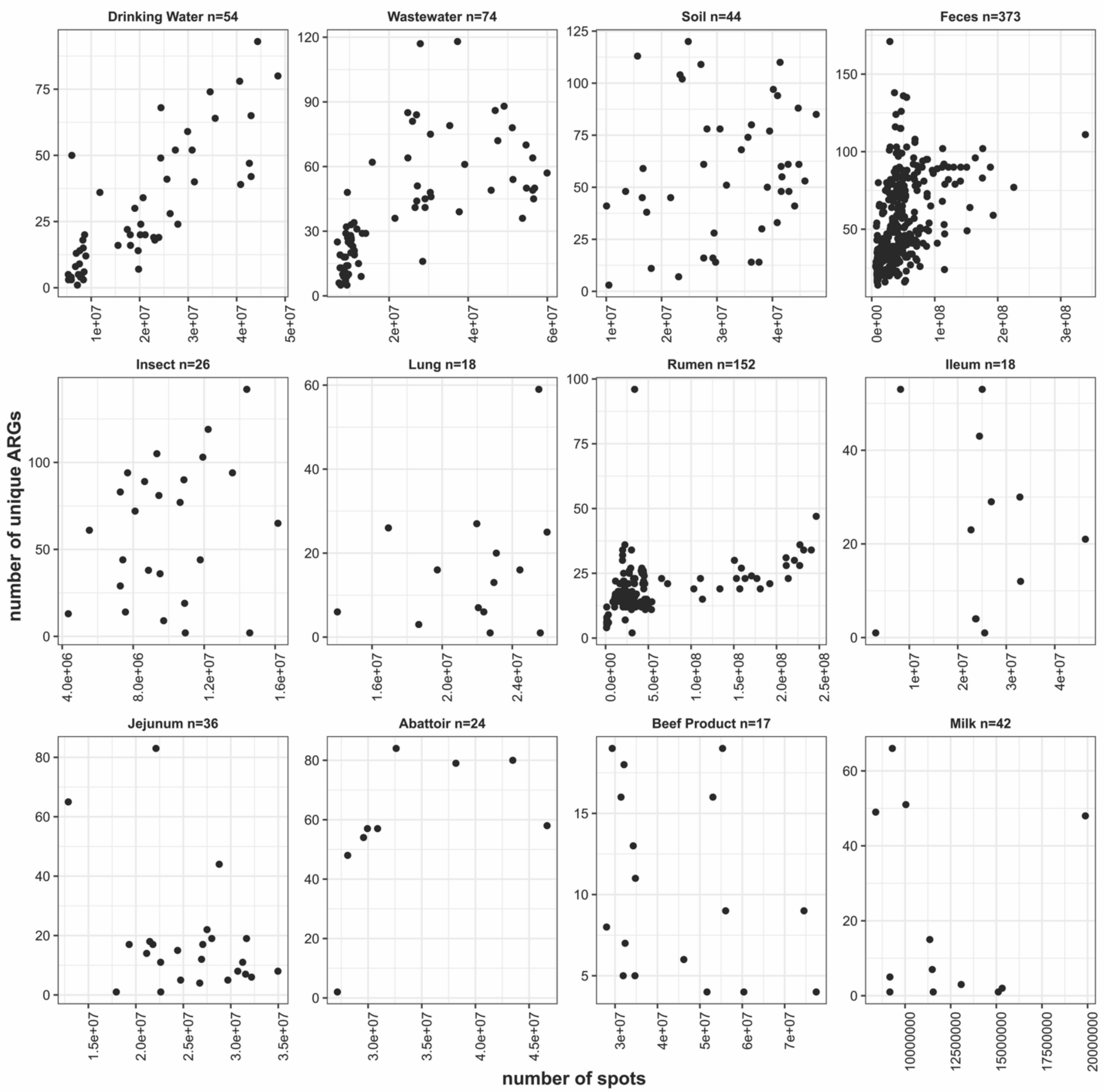

Figure S1. Plots of unique ARGs identified compared to sequence data within each SRR. The data is organized into panels by metagenome type. SRR spots are plotted to represent the data generated by individual experiments.

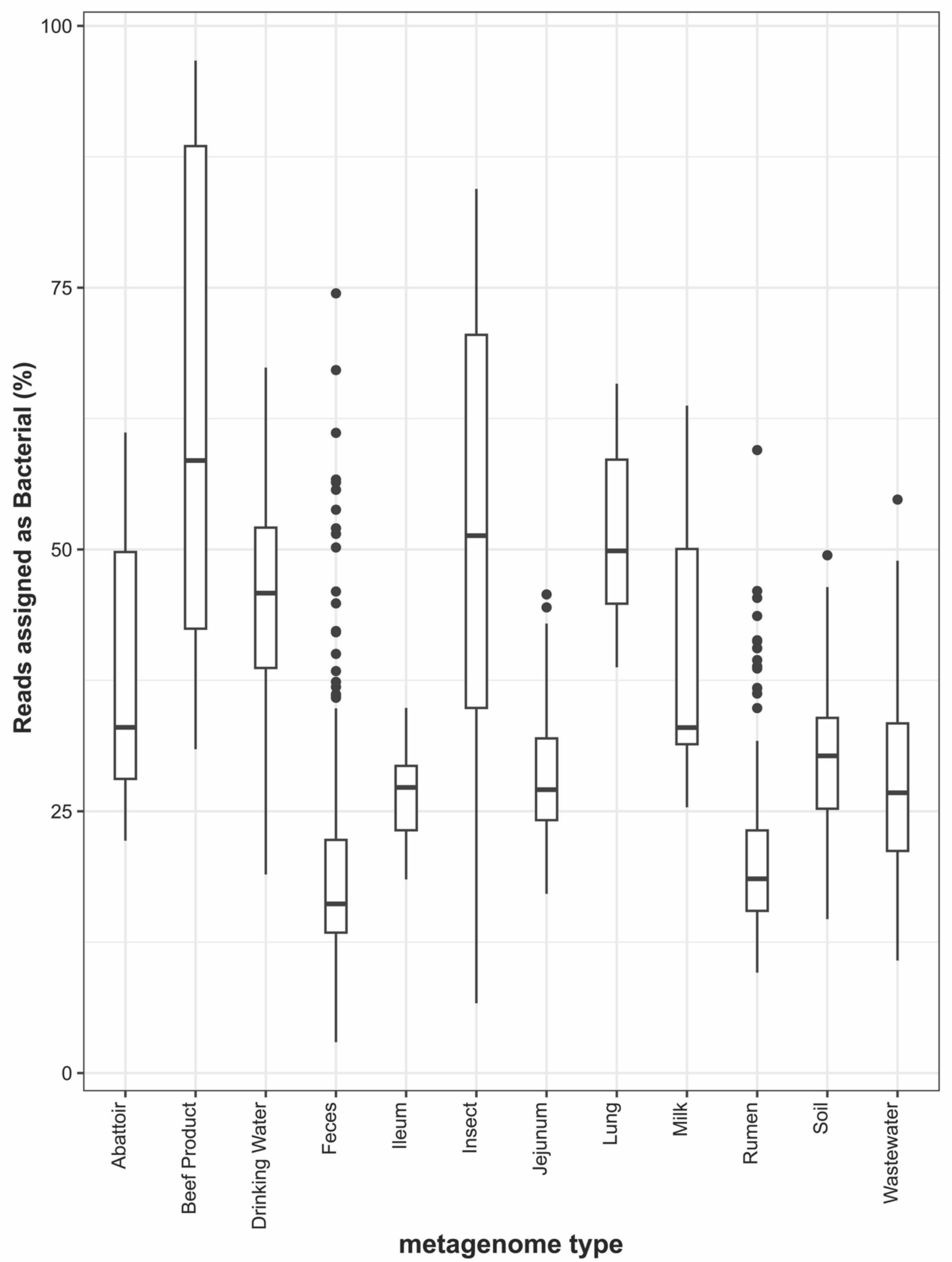

Figure S2. Bar plot showing the percentage of reads mapped to Bacteria by metagenome type. The plots show the average and distribution of bacterial reads identified in each individual metagenome type.

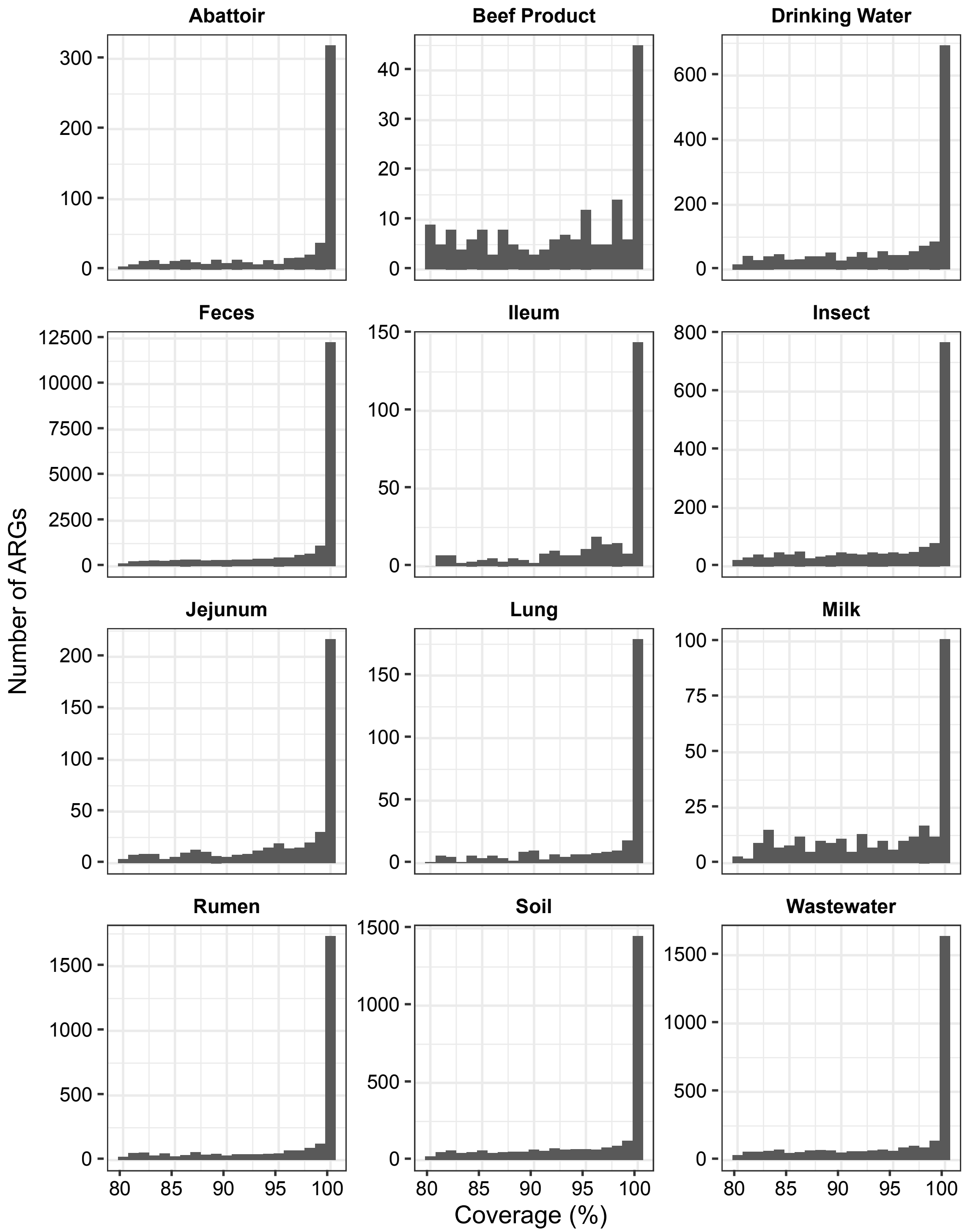

Figure S3. Histograms showing the number and distribution of ARGs identified in this study. DNA sequence reads for the vast majority of ARGs there were identified from each metagenome could be mapped to the full sequence of genes in the CARD v3.3.0.
